# Supplementary material for: Quantifying the trade-offs between renewable energy visibility and system costs
Source: Nat Commun. 2025 Apr 24;16:3853. doi: 10.1038/s41467-025-59029-1 (PMC12022082; doi:10.1038/s41467-025-59029-1)
Supplement: Supplementary file 1 — Supplementary Information [file 41467_2025_59029_MOESM1_ESM.pdf]

Supplementary Information

Quantifying the trade-offs between renewable energy visibility and system costs

Tsamara Tsani[1,2*†], Tristan Pelser[1,2], Romanos Ioannidis[3], Rachel Maier[1,2], Ruihong Chen[4], Stanley Risch[1,2], Felix Kullmann[1], Russell McKenna[4,5], Detlef Stolten[1,2], Jann Michael Weinand[1†]

[1]Institute of Climate and Energy Systems – Jülich Systems Analysis (ICE-2), Forschungszentrum Jülich GmbH, Jülich, 52425, Germany.
[2]Chair for Fuel Cells, RWTH Aachen University, 52062, Germany.
[3]Department of Architecture, Built Environment, and Construction Engineering, Politecnico di Milano, Milano, 20133, Italy.
[4]Chair of Energy Systems Analysis, ETH Zürich, 8092, Switzerland.
[5]Laboratory for Energy System Analysis, Paul Scherer Institute, Villigen, 5253, Switzerland.

*Corresponding author(s). E-mail(s): t.tsani@fz-juelich.de;
Contributing authors: t.pelser@fz-juelich.de; romanos.ioannidis@polimi.it; ra.maier@fz-juelich.de; ruchen@ethz.ch; stanley.risch@rwth-aachen.de; f.kullmann@fz-juelich.de; russell.mckenna@psi.ch; d.stolten@fz-juelich.de; j.weinand@fz-juelich.de;
†These authors contributed equally to this work.

Contents

Supplementary Table 1 2
Supplementary Figure 1 3
Supplementary Figure 2 4
Supplementary Figure 3 5
Supplementary Figure 4 6
Supplementary Figure 5 7
Supplementary Figure 6 8
Supplementary Figure 7 9
Supplementary Figure 8 10
Supplementary Figure 9 11
Supplementary Figure 10 12
Supplementary Figure 11 13
Supplementary Figure 12 14
Supplementary Figure 13 15
Supplementary Figure 14 16
Supplementary Figure 15 17
Supplementary References 18

Supplementary Table 1 Land exclusions for wind turbines and open-field PV (OFPV) based on Risch et al., 2022[1]. The Digital Basic-Landscape model (Basis-DLM) contains topographic objects located in an area in vector formats. Several geospatial locations of objects are also retrived from OpenStreetMap (OSM). SQR stands for Soil Quality Rate. CONTIS stands for Continental Shelf Information System. BGR stands for German Federal Institute for Geosciences and Natural Resources. WDPA stands for The World Database on Protected Areas. BFN stands for German Federal Agency for Nature Conservation.

**Constraint category**	**Land category**	**Buffer Wind [m]**	**Buffer OFPV [m]**	**Datasource**
a. Infrastructure				
Human settlements	"Inner" Residential Areas	1000	0	
	Residential	390	0	House perimeter[2]
	Mixed Buildings	390	10	
	Medical buildings	1000	10	
Airports & Radar	Airports	6000	-	Basis-DLM[3]
	Airfields	1500	-	
	Radio Navigation (VOR)	15000	-	OSM[4]
	Doppler VOR	10000	-	
Roads & Railways	Primary roads	194	22.5	Basis-DLM[3]
	Secondary & Regional Roads	174	22.5	
	Motorways	214	SQR>40, 15	Soil Quailty Rate[5]
	Railway	448	SQR>40, 15	
Energy Infrastructure	Power Lines	448	20	Basis-DLM[3]
	High voltage and data cables	500 (offshore)	-	CONTIS facilities
	Pipeline and platforms	500 (offshore)	-	
Industry & Commerce	Industrial & Commercial Areas	260	0	Basis-DLM[3]
	Mining Sites	0	0	
Military & Security	Military Zones	0	0	Basis-DLM[3]
	Land borders	100(on-)/15000(offshore)	100	Basis-DLM[6]
	Sea borders	500 (offshore)	-	Risch et al., 2022[1]
	Seismic Stations	1000	-	BGR[7]
	Sea depth	1000	-	Inspire Geoportal[8]
Culture & Leisure	Camp Sites		10	
	Recreational Areas		0	Basis-DLM[3]
	Cemeteries		0	
b. Geographical and technical				
Terrain	Slope	17 °	10 °	Copernicus[9]
Water Bodies	Lakes	100	10	Basis-DLM[3]
	Rivers	100	10	
	Streams	0	10	OSM[4]
c .Conservation				
Nature	Protected Natural Areas	300	0	
	Protected Water Zones	50	-	
	National Parks	300	0	WDPA[10]
	Bird Protected Areas	300(on-)/0(offshore)	0	
	Biospheres	0	-	BFN[11]
	Farmland	-	200	
Cultural	Historical Sites	1000	0	OSM[4]

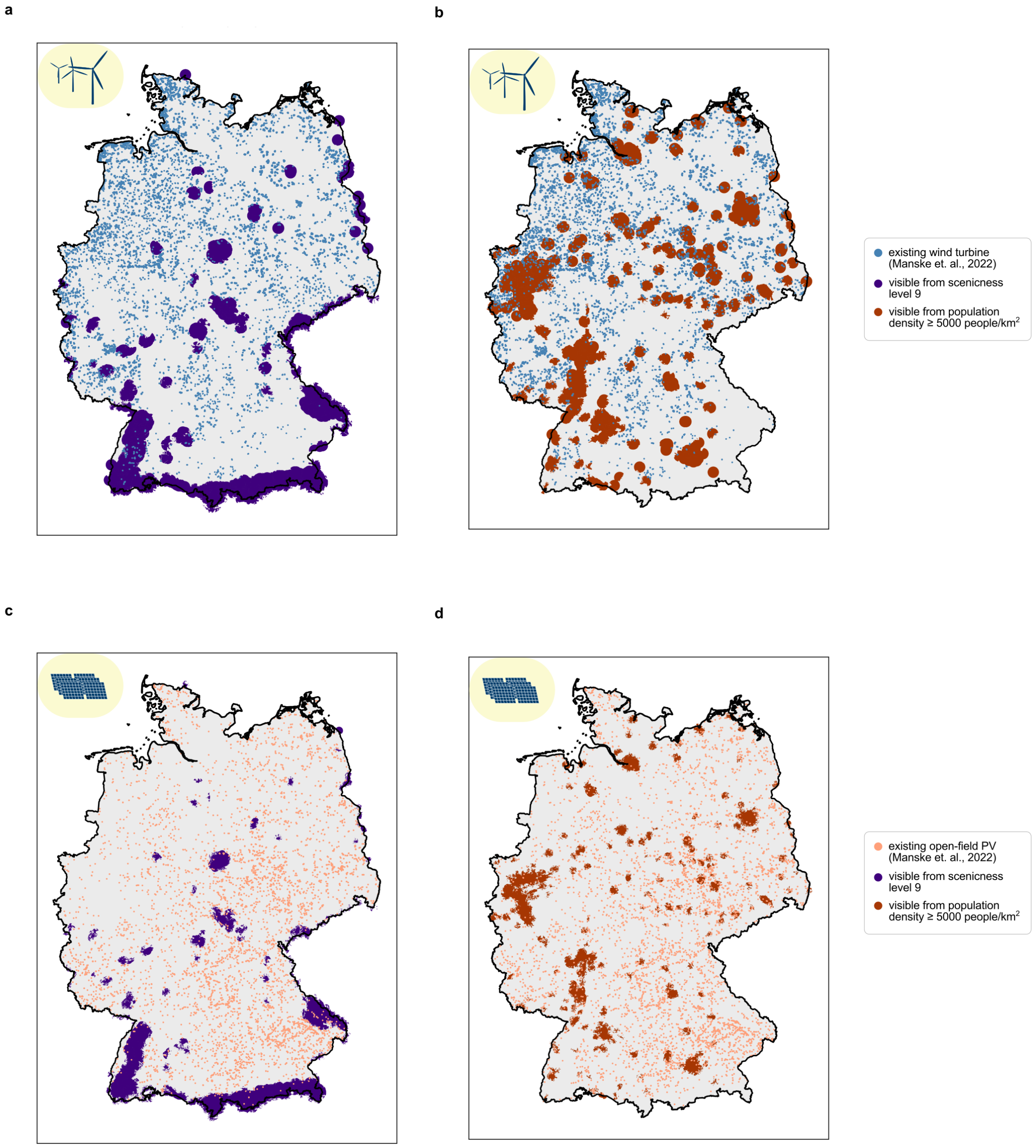

Supplementary Figure 1 The visibility of existing onshore wind turbines and open-field photovoltaics (PV) projects from scenic or densely populated areas. The figure overlay the locations of existing wind turbines and open-field PV with visibility map from (**a, c**) scenicness level = 9 and from areas with (**b, d**) population density $\geq$ 5000 people per km^2, respectively. A mere 3% of existing onshore wind turbines and 2% of open-field PV installations are visible from the most scenic areas. Conversely, 12% of the existing onshore wind turbines and 2% of open-field PV installations are visible from densely populated areas. Similar analyses were also conducted for all other visibility threshold scenarios to analyze the visibility of the currently existing renewable infrastructures.

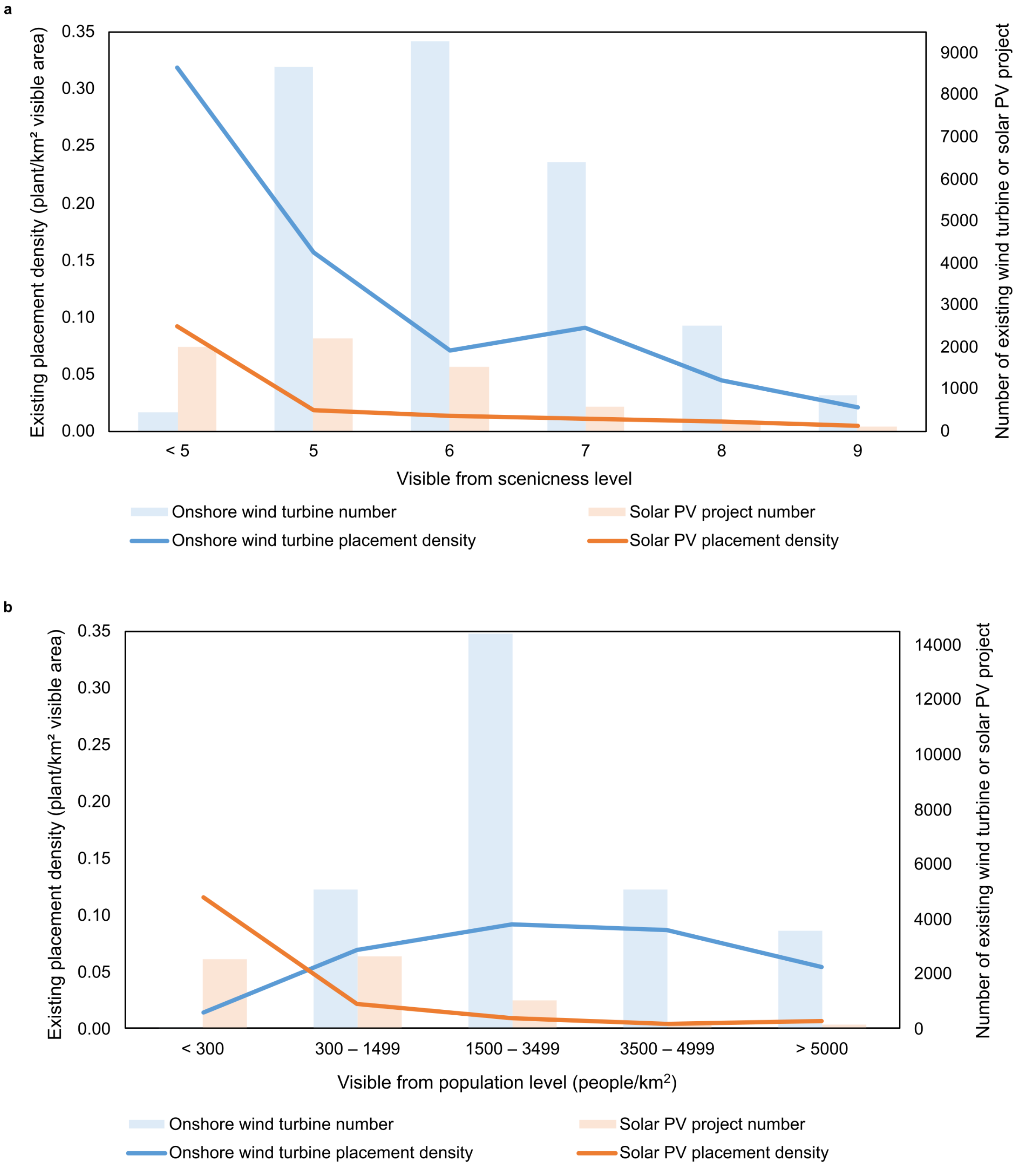

Supplementary Figure 2 Concentration of the existing renewable energy plants by their visibility from different scenicness (a) and population density levels (b). The placement density (in line graphs) is calculated by dividing the number of existing wind turbine or open-field photovoltaics (PV) projects that are visible from different scenicness and population density levels by the reverse-viewshed areas (in km^2) generated from viewpoints with the respective scenicness or population density levels. In subfigure a, there is a relatively high concentration of onshore wind turbines and open-field PV that are visible from areas with low scenicness levels (< 5), but not visible from areas with scenicness level ≥ 5. As the scenicness level increases, the concentration of these renewable energy plants decreases. For population density, this trend is only visible for open-field PV.

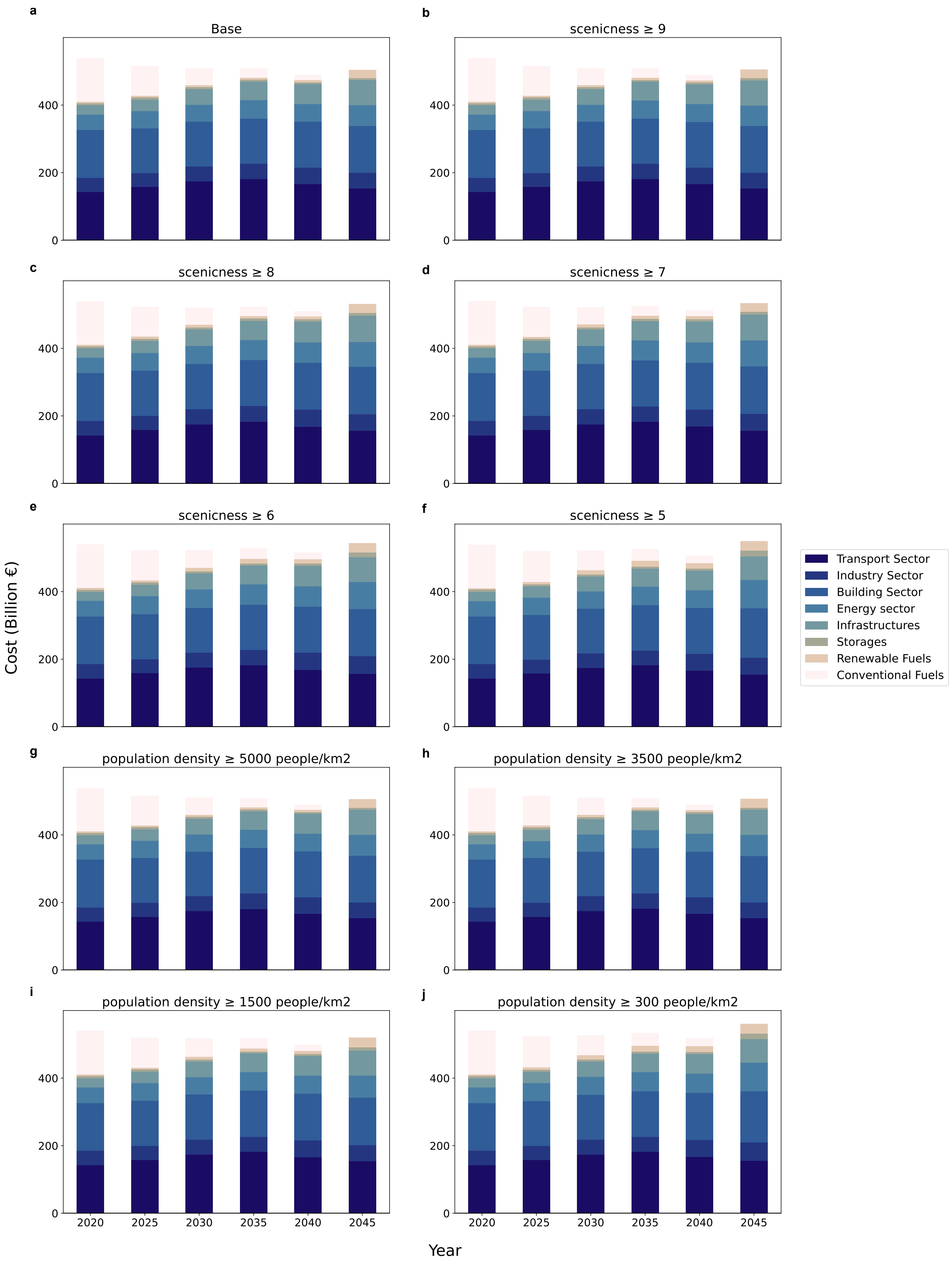

Supplementary Figure 3 Overall system cost by sector and major energy-related sub-sectors across visibility scenarios. Costs at (**a**) the base scenario, at scenario where renewable energy are not visible from (**b**) scenicness ≥ 9, (**c**) scenicness ≥ 8, (**d**) scenicness ≥ 7, (**e**) scenicness ≥ 6, (**f**) scenicness ≥ 5, (**g**) population density ≥ 5000 people per km^2, (**h**) ≥ 3500 people per km^2, (**i**) ≥ 1500 people per km^2, and (**j**) ≥ 300 people per km^2. The energy sector accounts for domestic energy supply. The infrastructure sector accounts for grid costs. Renewable and conventional fuels represent the cost for imported fuels.

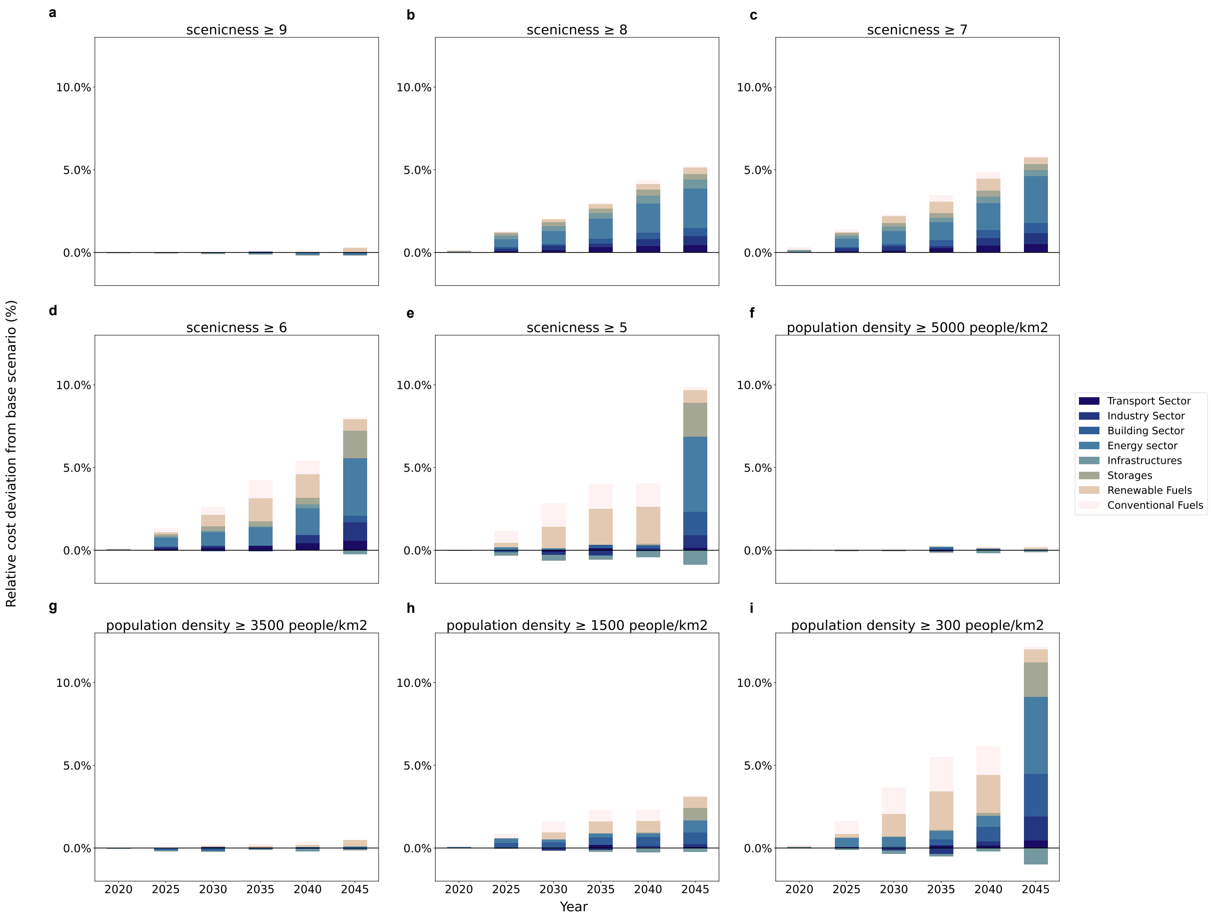

Supplementary Figure 4 Relative deviation of overall system cost by sector and major energy-related sub-sectors across scenarios. The subfigures show relative deviation of overall system cost at scenario where renewable energy are not visible from (**a**) scenicness ≥ 9, (**b**) scenicness ≥ 8, (**c**) scenicness ≥ 7, (**d**) scenicness ≥ 6, (**e**) scenicness ≥ 5, (**f**) population density ≥ 5000 people per km^2, (**g**) ≥ 3500 people per km^2, (**h**) ≥ 1500 people per km^2, and (**i**) ≥ 300 people per km^2. The energy sector accounts for domestic energy supply. The infrastructure sector accounts for grid costs. Renewable and conventional fuels represent the cost for imported fuels.

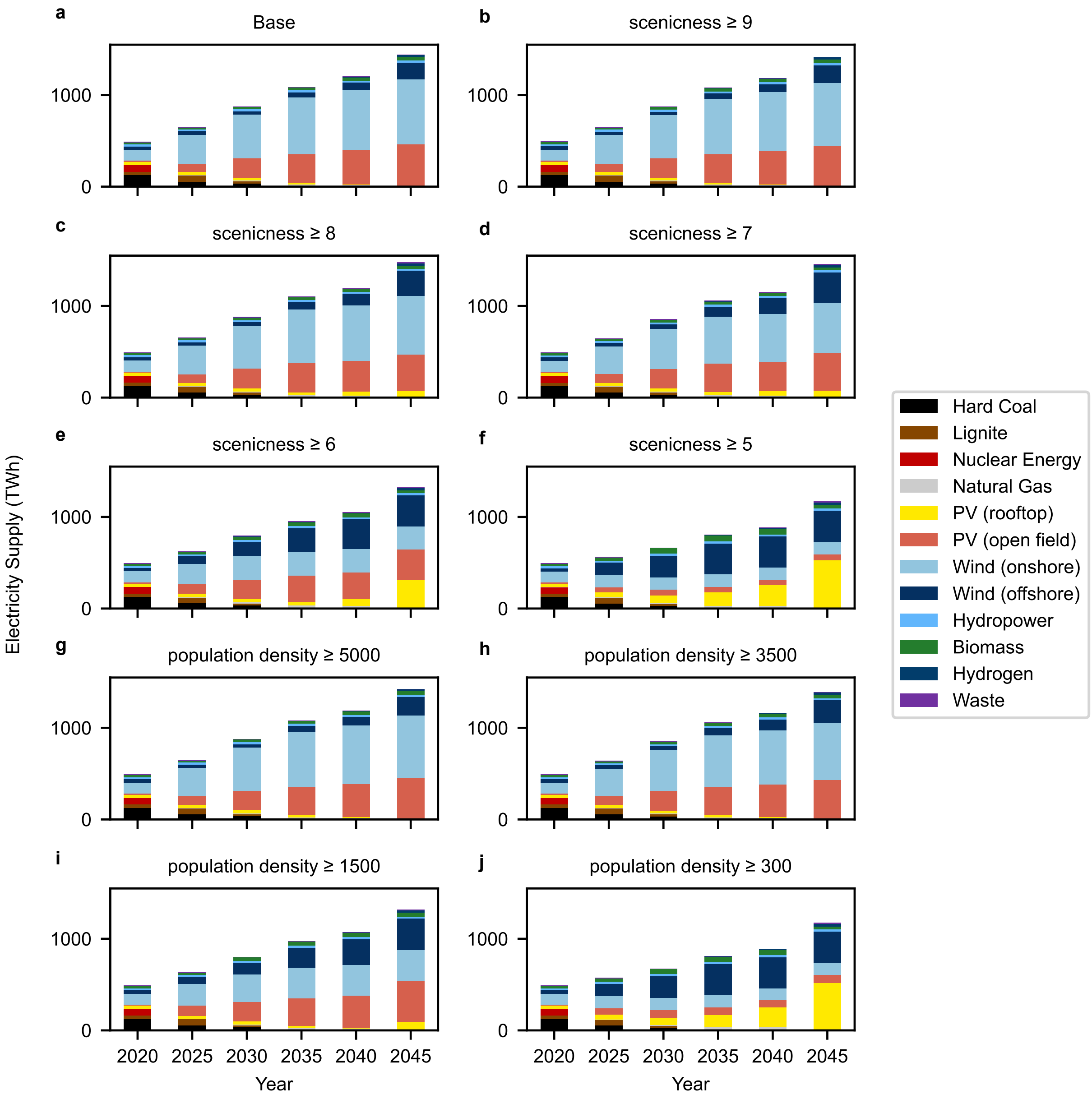

Supplementary Figure 5 Electricity supplies by sources across visibility scenarios. a At the base scenario, onshore wind power and open-field photovoltaics (PV) dominates the future electricity supply. As the visibility restrictions progress, the supply from open-field PV and onshore wind is gradually replaced by rooftop PV and offshore wind power. The changes in composition of electricity supply sources are shown in the subfigures at scenario where renewable energy are not visible from (**b**) scenicness ≥ 9, (**c**) scenicness ≥ 8, (**d**) scenicness ≥ 7, (**e**) scenicness ≥ 6, (**f**) scenicness ≥ 5, (**g**) population density ≥ 5000 people per km^2, (**h**) ≥ 3500 people per km^2, (**i**) ≥ 1500 people per km^2, and (**j**) ≥ 300 people per km^2.

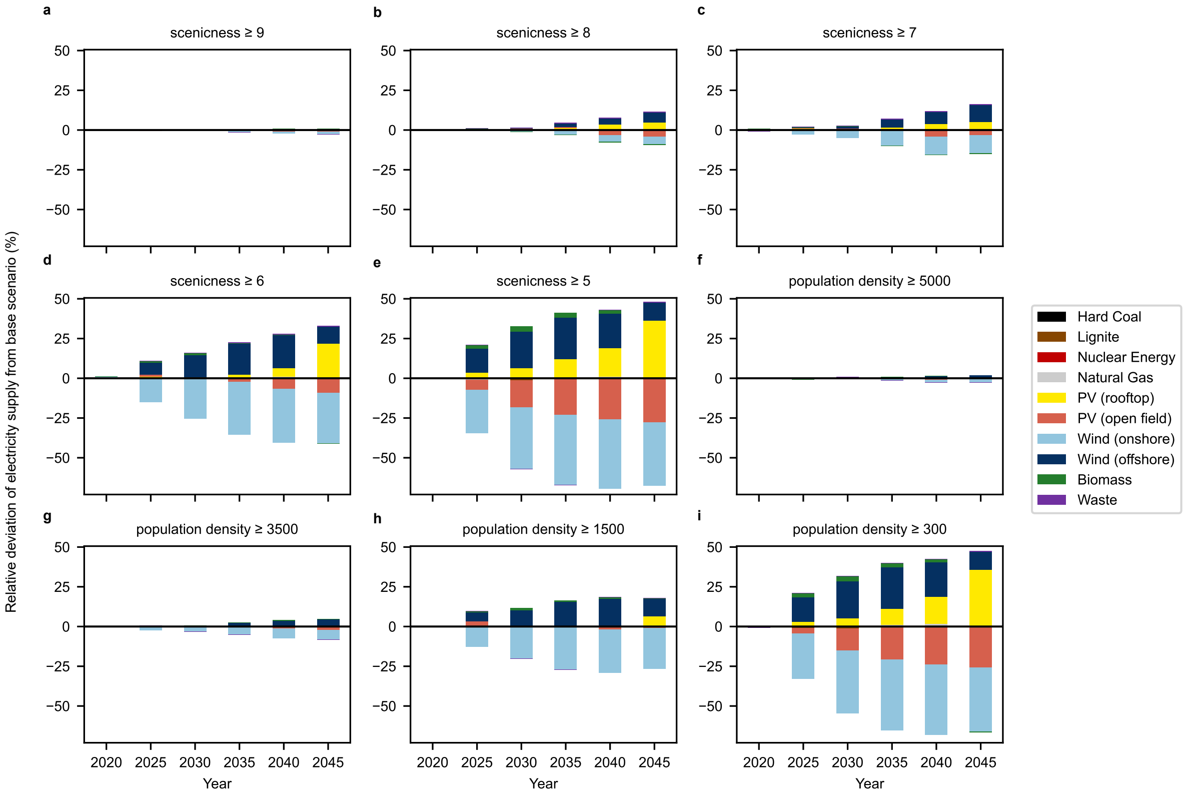

Supplementary Figure 6 Relative deviation of electricity supplies by sources across visibility scenarios. Excluding onshore wind and open-field photovoltaics (PV) that are visible from the most scenic (scenicness = 9) or densely populated areas (population density $\geq$ 3500 people per km^2) does not affect the optimal system cost. The subfigures show relative deviation of electricity supplies by sources at scenario where renewable energy infrastructures are not visible from (**a**) scenicness $\geq$ 9, (**b**) scenicness $\geq$ 8, (**c**) scenicness $\geq$ 7, (**d**) scenicness $\geq$ 6, (**e**) scenicness $\geq$ 5, (**f**) population density $\geq$ 5000 people per km^2, (**g**) $\geq$ 3500 people per km^2, (**h**) $\geq$ 1500 people per km^2, and (**i**) $\geq$ 300 people per km^2.

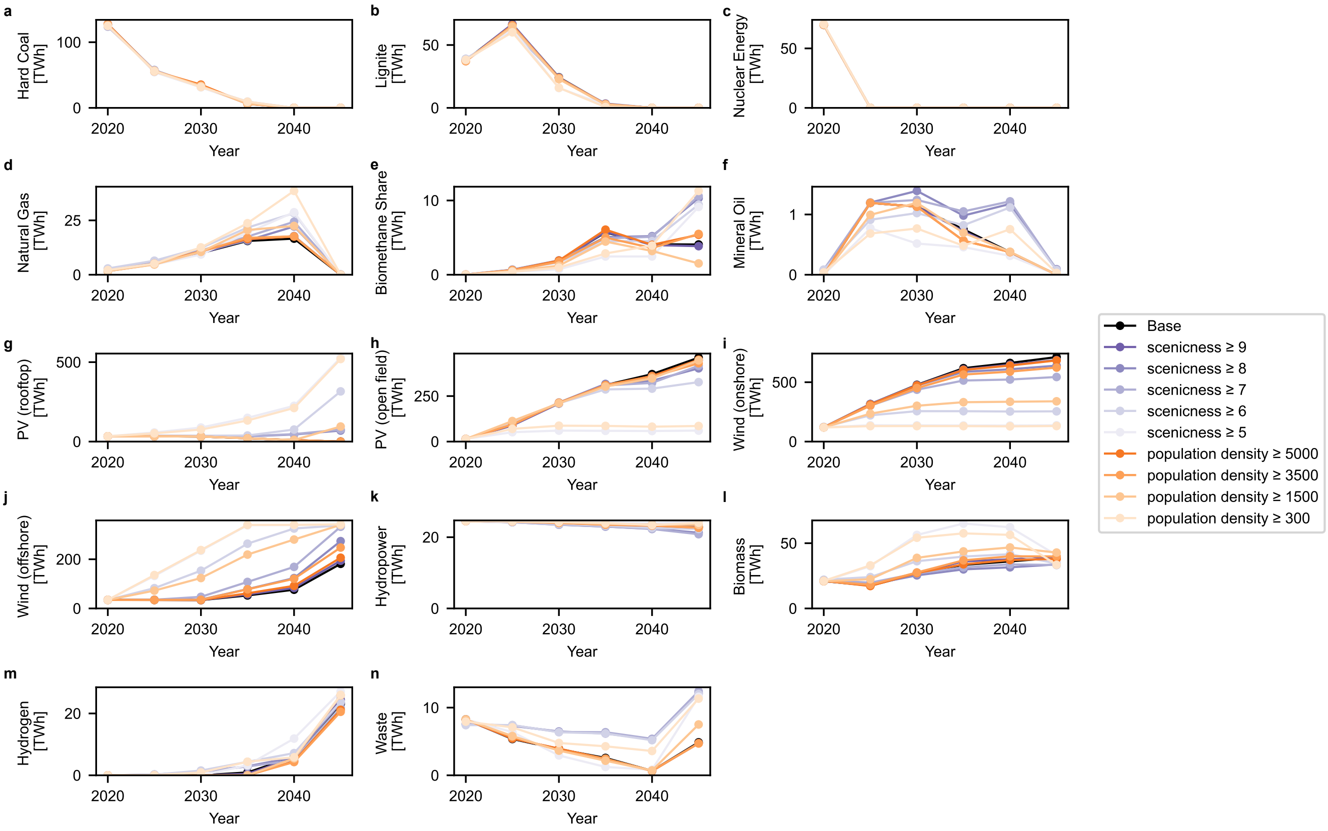

Supplementary Figure 7 Changes in electricity supply by sources across visibility scenarios. Stricter restrictions on the visibility of large-scale onshore wind and open-field photovoltaics (PV) causes supply from these technologies to decrease (seen from subfigures **i** and **h** at light purple and light orange color lines). The future electricity supply from those technologies are substituted by other sources, namely wind offshore (**j**), rooftop PV (**g**), waste (**n**), and biomass (**l**). At these strict visibility scenarios, there is an increases in fossil-fuel based supplies (**b,d,f**) and reaches their peak in 2040 to ensure the attainment of greenhouse gas (GHG) neutral targets. The figure shows changes in electricity supply by hard coal (**a**), lignite (**b**), nuclear energy (**c**), natural gas (**d**), biomethane (**e**), mineral oil (**f**), rooftop PV (**g**), open-field PV (**h**), onshore wind (**i**), offshore wind (**j**), hydropower (**k**), biomass (**l**), hydrogen (**m**), and waste (**n**) across visibility scenarios.

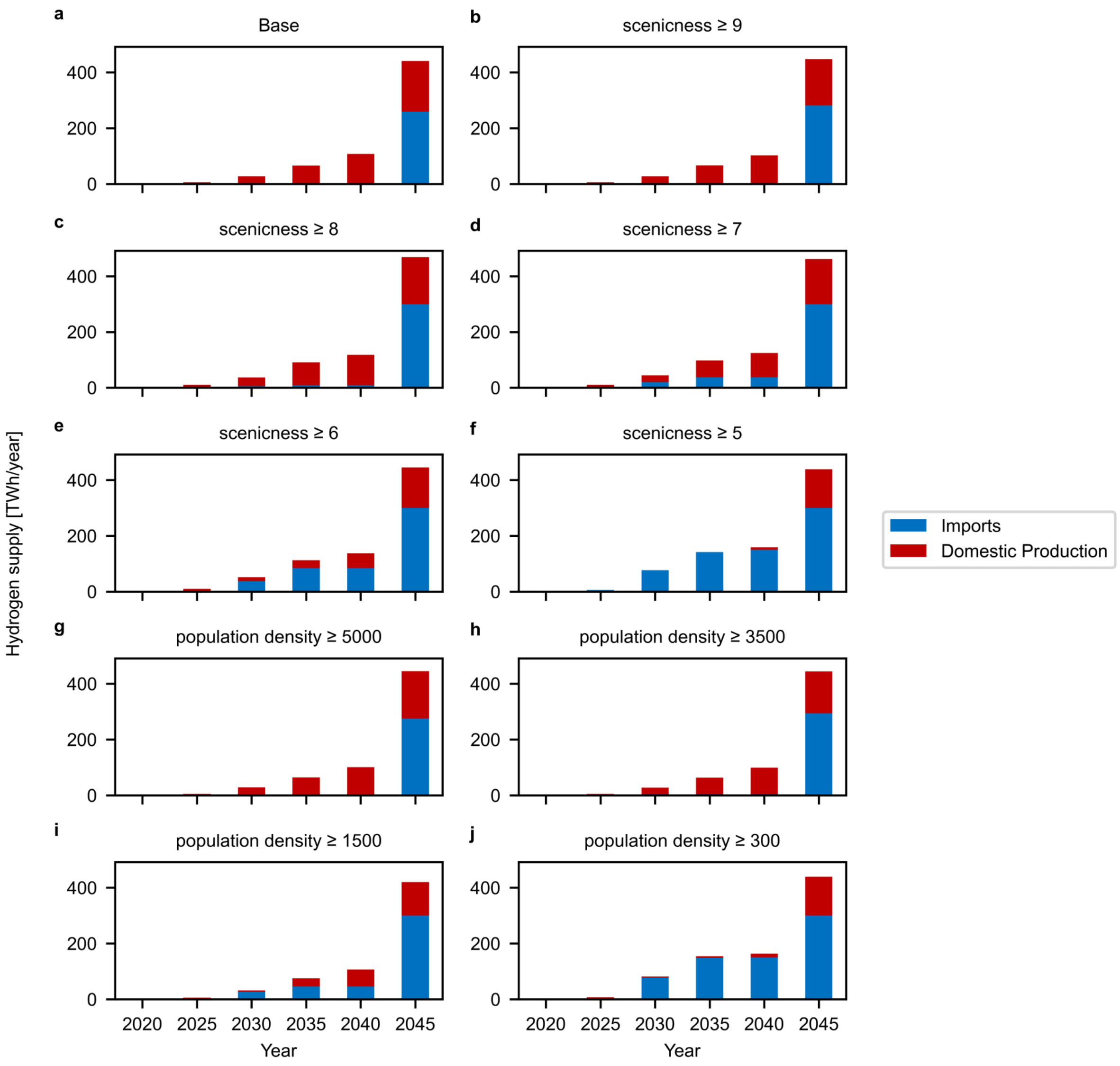

Supplementary Figure 8 Comparison of hydrogen supply route across visibility scenarios. At strict visibility restrictions of large-scale onshore wind and open-field photovoltaics (PV), i.e., at scenario scenicness ≥ 5 (**f**) and population density ≥ 300 people per km^2 (**j**), hydrogen demand is predominantly supplied by imports. In contrast to the base (**a**) and moderate visibility restrictions (**b,c,g,h**), where hydrogen imports is only required from 2045. The figure shows the share of hydrogen supply routes at the base scenario (**a**), at scenarios where renewable energy infrastructures are not visible from (**b**) scenicness ≥ 9, (**c**) scenicness ≥ 8, (**d**) scenicness ≥ 7, (**e**) scenicness ≥ 6, (**f**) scenicness ≥ 5, (**g**) population density ≥ 5000 people per km^2, (**h**) ≥ 3500 people per km^2, (**i**) ≥ 1500 people per km^2, and (**j**) ≥ 300 people per km^2.

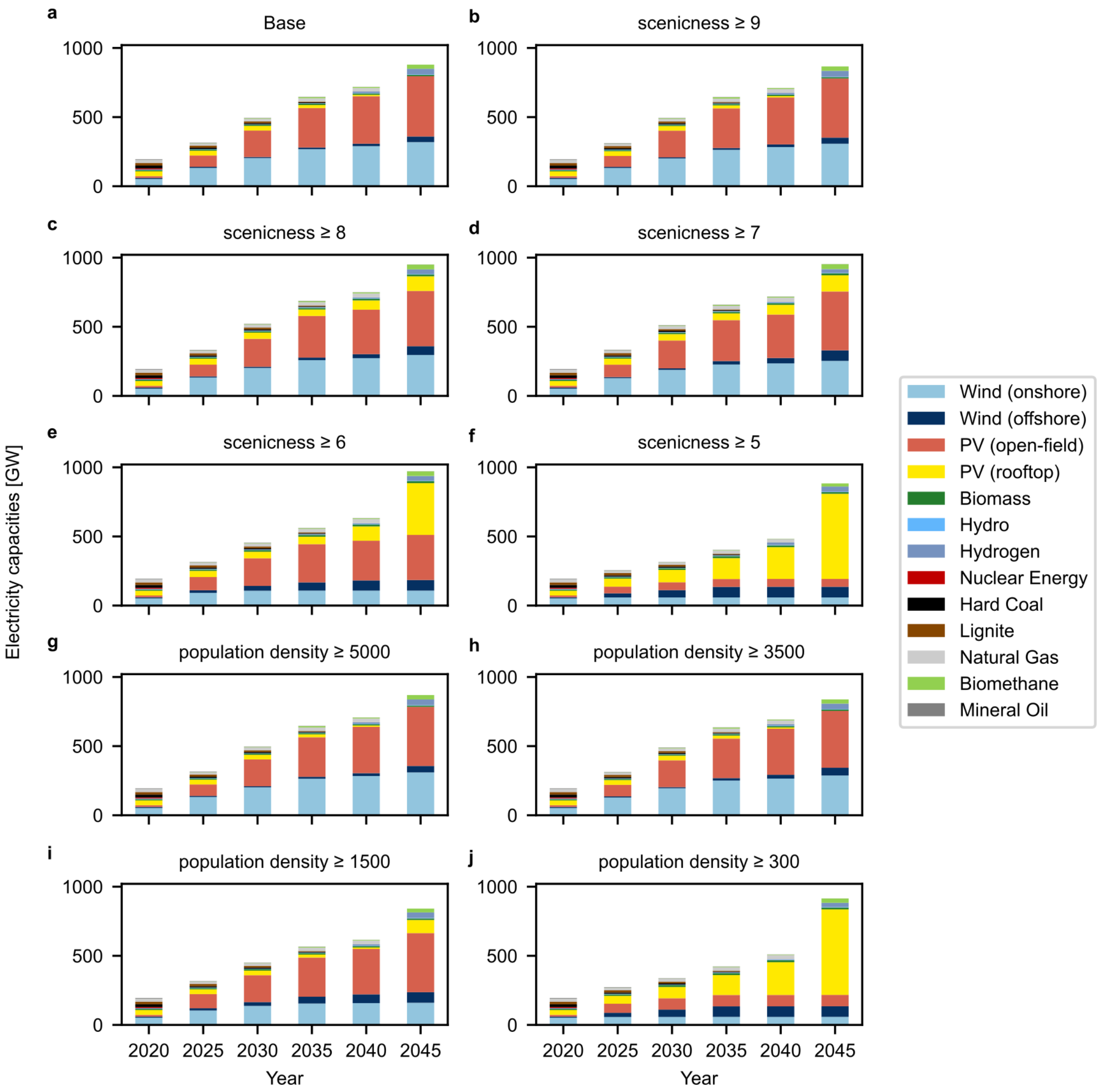

Supplementary Figure 9 Electricity capacity by sources across visibility scenarios. At the base (**a**) and moderate visibility restrictions (**b,c,d,g,h,i**), the future electricity systems are dominated by installation of open-field photovoltaics (PV) and onshore wind power. As the visibility restrictions progress, massive installment of rooftop PV is required (**e,f,j**).

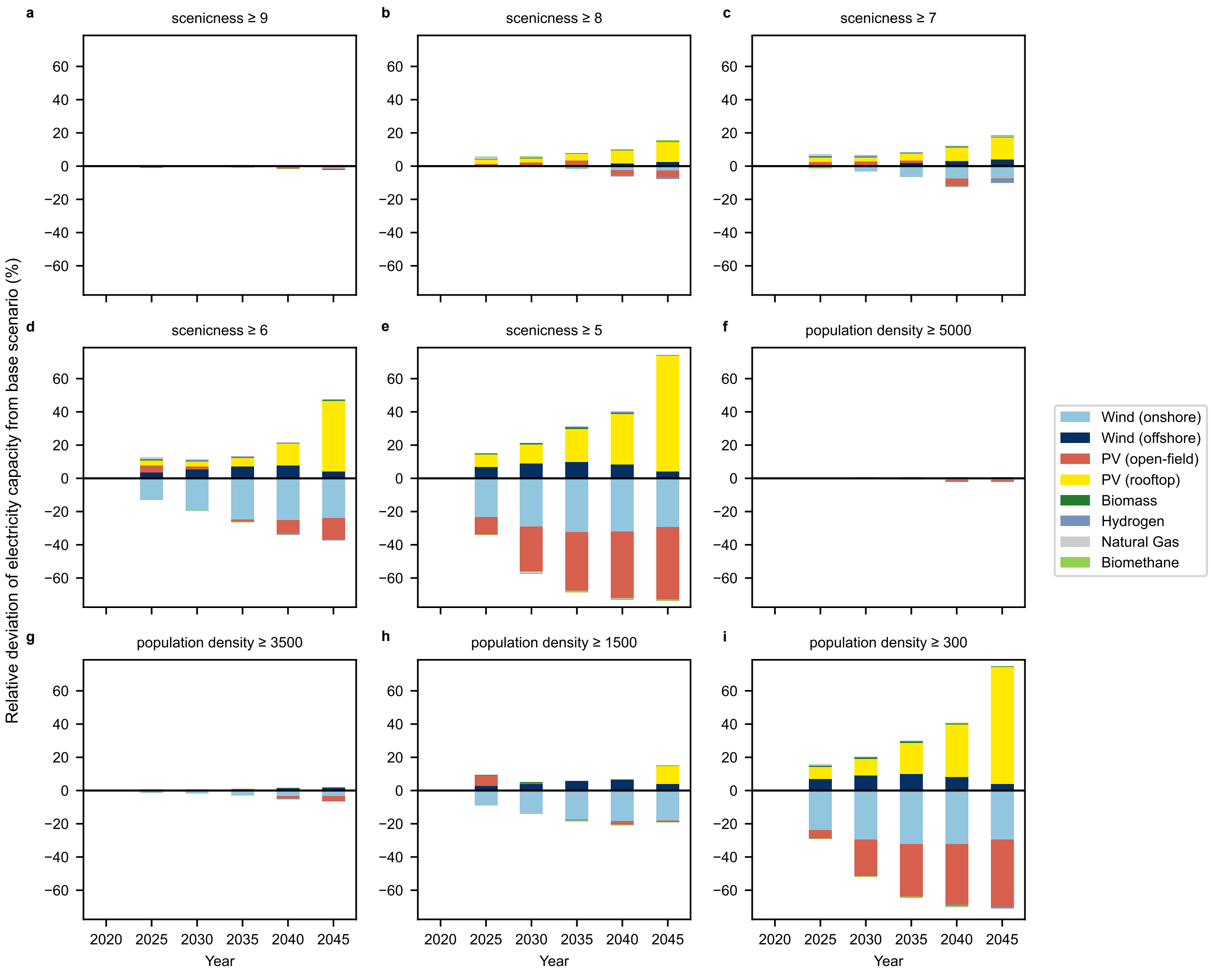

Supplementary Figure 10 Relative deviation of electricity capacity from the base scenario. This figure demonstrates changes of installed capacity at each visibility restriction scenario, compared to the base scenario. Minimizing visibility of large-scale onshore wind and open-field PV would shift the capacity investment to rooftop photovoltaics (PV) and offshore wind power. The figure shows relative deviation of electricity capacity at scenario where renewable energy are not visible from (**a**) scenicness ≥ 9, (**b**) scenicness ≥ 8, (**c**) scenicness ≥ 7, (**d**) scenicness ≥ 6, (**e**) scenicness ≥ 5, (**f**) population density ≥ 5000 people per km^2, (**g**) ≥ 3500 people per km^2, (**h**) ≥ 1500 people per km^2, and (**i**) ≥ 300 people per km^2.

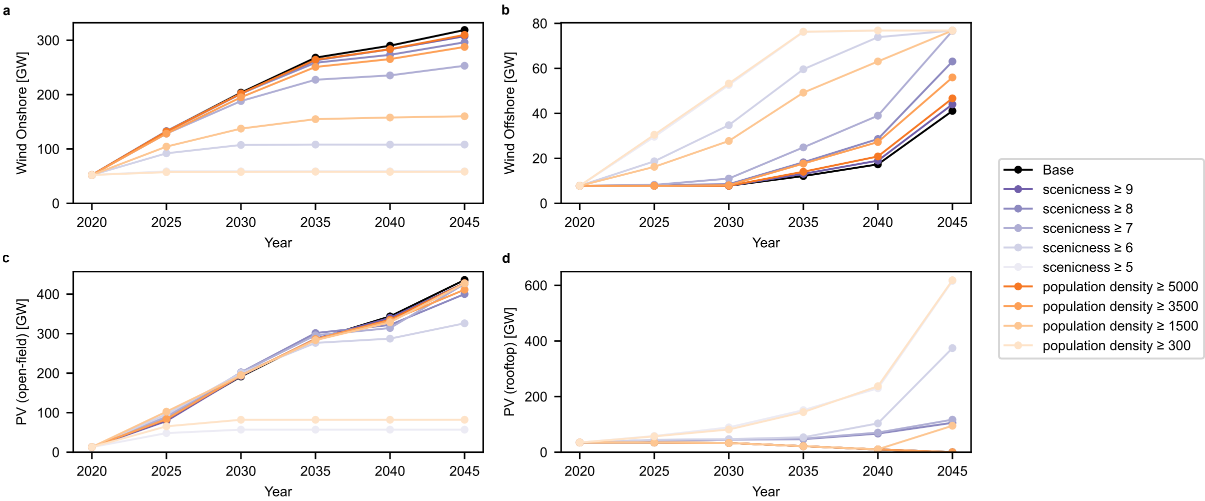

Supplementary Figure 11 Comparison of changes in renewable energy capacity across visibility scenarios. This figure demonstrates changes of installed capacity of onshore wind (**a**), offshore wind (**b**), open-field photovoltaics (PV) (**c**), and rooftop PV (**d**) across visibility scenarios. The lighter colors show stricter visibility restrictions for onshore wind and open-field PV.

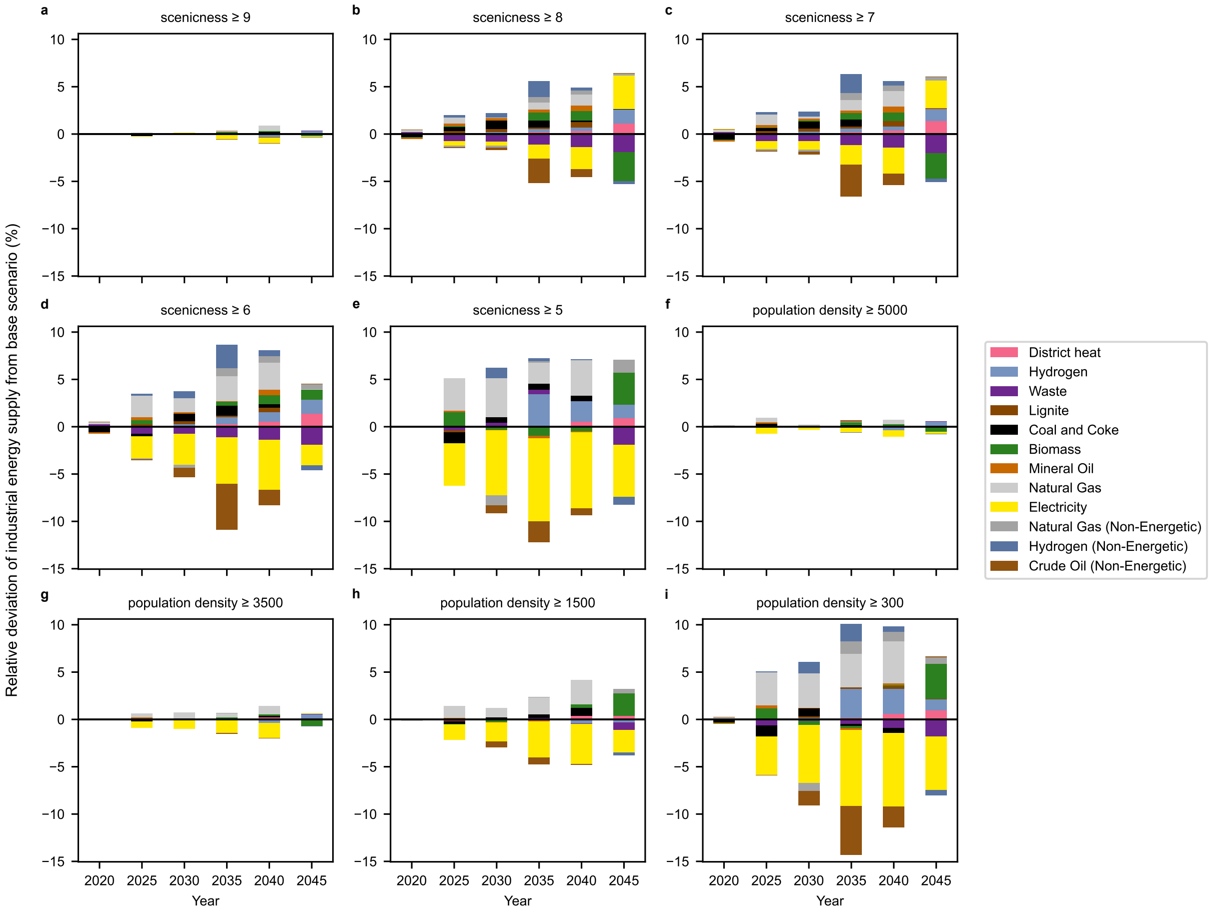

Supplementary Figure 12 Relative deviation of energy supply by sources from the base scenario in industrial sector. There is small to negligible changes in the energy supply by sources in industrial sector at scenarios where renewable energy are not visible only from the most scenic (**a**) or the most densely populated areas (**f**). As visibility restrictions progress, the supply from electricity reduces, and the industry sector starts to rely on fossil-fuel based sources or biomass. The figure shows relative deviation of energy supply by sources in industrial sector at scenario where renewable energy infrastructures are not visible from (**a**) scenicness ≥ 9, (**b**) scenicness ≥ 8, (**c**) scenicness ≥ 7, (**d**) scenicness ≥ 6, (**e**) scenicness ≥ 5, (**f**) population density ≥ 5000 people per km^2, (**g**) ≥ 3500 people per km^2, (**h**) ≥ 1500 people per km^2, and (**i**) ≥ 300 people per km^2.

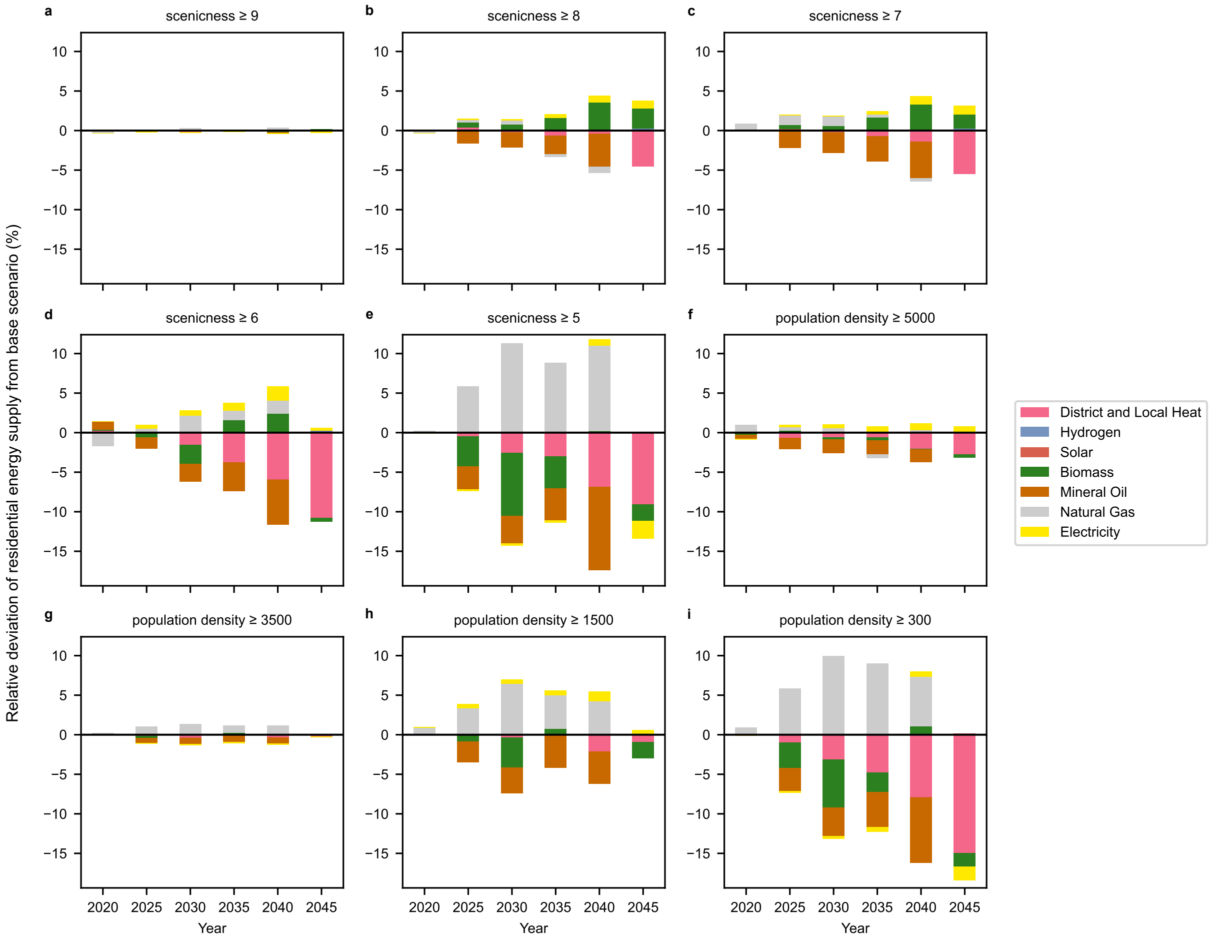

Supplementary Figure 13 Relative deviation of energy supply by sources from the base scenario in residential sector. There is small to negligible changes in the energy supply by sources in residential sector at scenarios where renewable energy are not visible only from the most densely populated areas (**f, g**) or the most scenic (**a**). As visibility restrictions progress, the supply from district and local heat reduces, and the industry sector starts to rely on natural gas (**e, h, i**). The figure shows relative deviation of energy supply by sources in residential sector at scenario where renewable energy infrastructures are not visible from (**a**) scenicness ≥ 9, (**b**) scenicness ≥ 8, (**c**) scenicness ≥ 7, (**d**) scenicness ≥ 6, (**e**) scenicness ≥ 5, (**f**) population density ≥ 5000 people per km^2, (**g**) ≥ 3500 people per km^2, (**h**) ≥ 1500 people per km^2, and (**i**) ≥ 300 people per km^2.

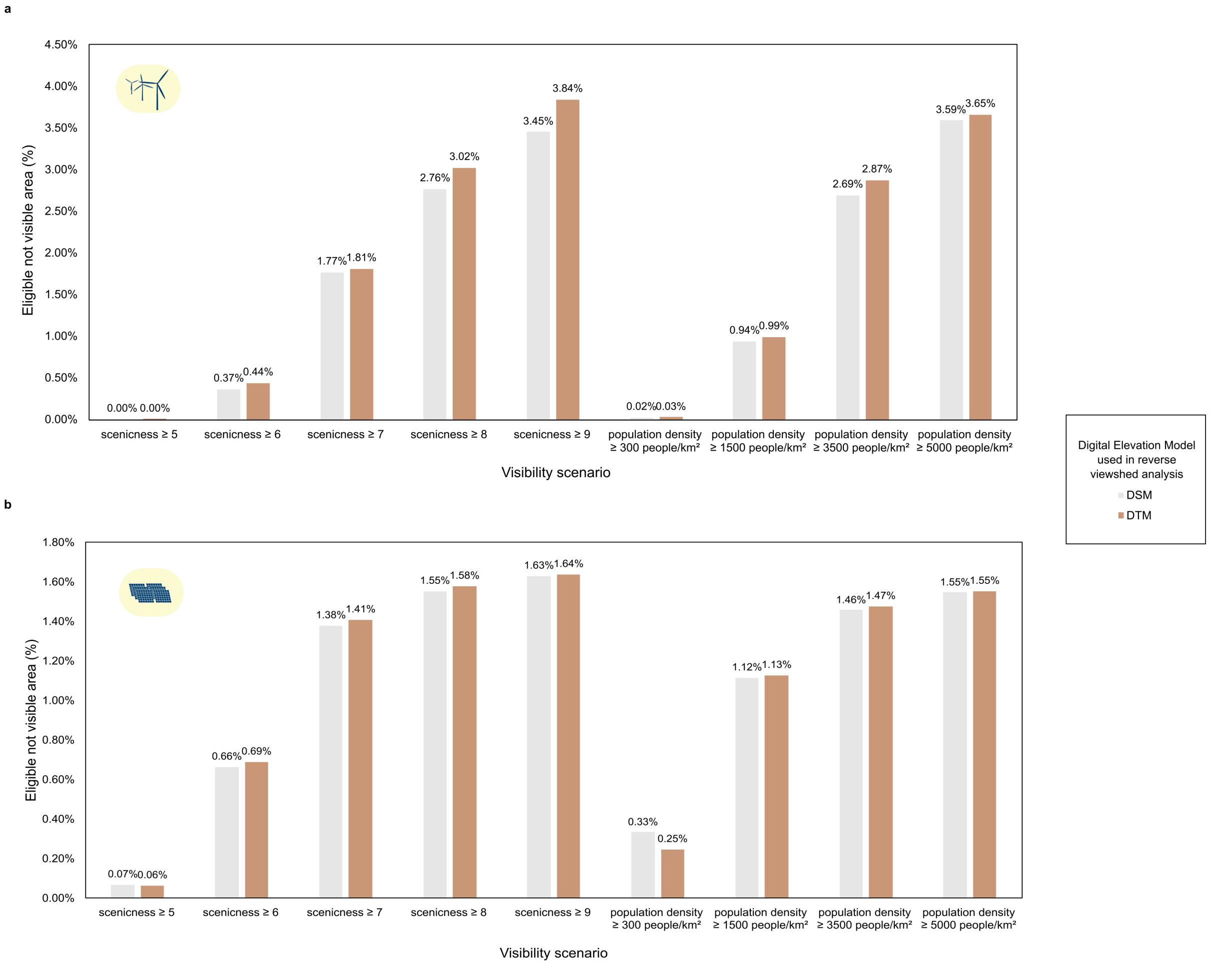

Supplementary Figure 14 Sensitivity analysis on the resulting eligible area in Sachsen, Germany based on different Digital Elevation Models (DEM) used in the reverse viewshed calculation. Deviation in the eligible, not visible area in Sachsen, Germany, for (**a**) onshore wind and (**b**) open-field photovoltaics (PV) placements at each scenario when using Digital Surface Model (DSM) with 25 m resolution, shown in grey color and Digital Terrain Model (DTM) with 20 m resolution, shown in brown color). The eligible area exhibits minimal variation of 0 – 0.5%.

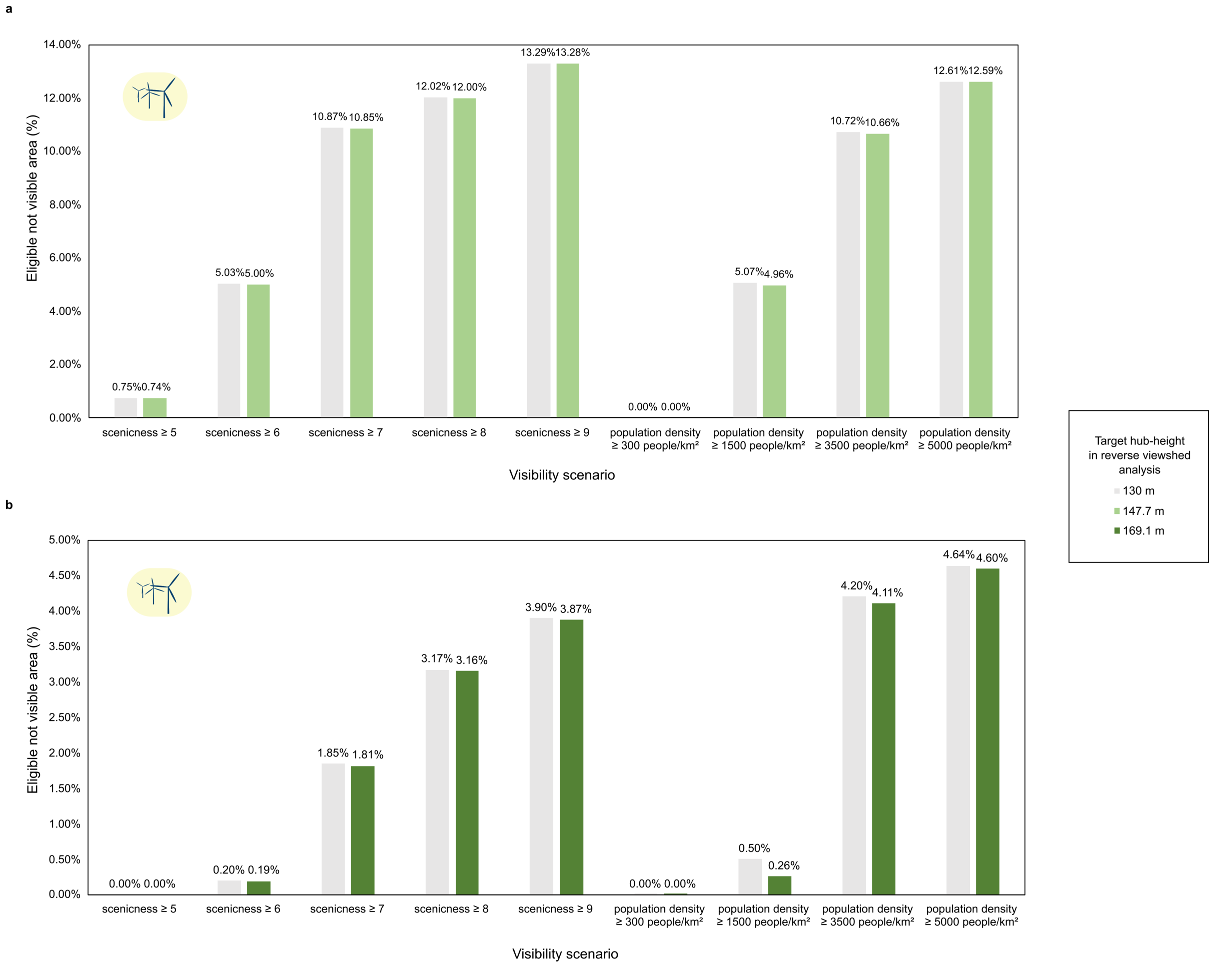

Supplementary Figure 15 Sensitivity analysis on the resulting eligible area for onshore wind placements based on different target hub heights used in the reverse viewshed calculation. Deviation in the eligible, not visible area at each scenario when using uniform hub-height (130 m, in grey) and optimal turbine hub-height in (**a**) Sachsen Anhalt (147.7 m, in light green) and (**b**) Baden Wurttemberg (169.1 m, in green) in the reverse viewshed analysis. The deviations in eligible not visible area are minimal (0 – 0.11% for Sachsen Anhalt and 0 – 0.24% for Baden Wurttemberg). The robustness of the results is maintained for optimal future turbine height. The unit m shown in the figure legend stands for meter.

Supplementary References

[1] Risch, S. *et al.* Potentials of renewable energy sources in germany and the influence of land use datasets. *Energies* **15** (2022).

[2] GeoBasis-DE / BKG. Geobasisdaten ©: Amtliche Hausumringe Deutschland (HU-DE) (2021). URL https://gdz.bkg.bund.de/index.php/default/amtliche-hausumringe-deutschland-hu-de.html.

[3] GeoBasis-DE / BKG. Geobasisdaten ©: Digitales Basis-Landschaftsmodell (Ebenen) (Basis-DLM) (2021). URL https://gdz.bkg.bund.de/index.php/default/digitales-basis-landschaftsmodell-ebenen-basis-dlm-ebenen.html.

[4] OpenStreetMap contributors. OpenStreetMap (2017). URL https://www.openstreetmap.org.

[5] Federal Institute for Geosciences and Natural Resources (BGR). Ackerbauliches ertragspotenzial der böden in deutschland 1:1.000.000. datenquelle: Sqr1000 v1.0 (2013).

[6] GeoBasis-DE / BKG. Geobasisdaten ©: Verwaltungsgebiete 1:250 000 (VG250) (2021). URL https://gdz.bkg.bund.de/index.php/default/digitales-basis-landschaftsmodell-ebenen-basis-dlm-ebenen.html.

[7] Bundesanstalt für Geowissenschaften und Rohstoffe (BGR). Informationen zu deutschen Seismometer-Stationen (2021). URL https://www.bgr.bund.de/.

[8] Bundesamt für Seeschifffahrt und Hydrographie (BSH). Höhe (Bathymetrie) - INSPIRE-Download-Service (2018). URL https://inspire-geoportal.ec.europa.eu/srv/api/records/6fe1bb6a-c915-45fe-b84c-d88e4aec55c1?language=ger.

[9] Copernicus. EU-DEM v1.1 (2016). URL https://www.eea.europa.eu/en/datahub/datahubitem-view/d08852bc-7b5f-4835-a776-08362e2fbf4b.

[10] UNEP-WCMC, IUCN. The world database on protected areas (2016). URL https://www.protectedplanet.net/.

[11] Bundesamt für Naturschutz. BfN-Datensatz (2021). URL https://www.bfn.de/thema/karten-und-daten.
